# A versatile contribution of both aminopeptidases N and ABC transporters to Bt Cry1Ac toxicity in the diamondback moth

**DOI:** 10.1186/s12915-022-01226-1

**Published:** 2022-02-04

**Authors:** Dan Sun, Liuhong Zhu, Le Guo, Shaoli Wang, Qingjun Wu, Neil Crickmore, Xuguo Zhou, Alejandra Bravo, Mario Soberón, Zhaojiang Guo, Youjun Zhang

**Affiliations:** 1 Department of Plant Protection, Institute of Vegetables and Flowers, Chinese Academy of Agricultural Sciences, Beijing, 100081 China; 2 Guangdong Laboratory for Lingnan Modern Agriculture, Guangdong, 510642 China; 3 School of Life Sciences, University of Sussex, Brighton, BN1 9QE UK; 4 Department of Entomology, University of Kentucky, Lexington, KY 40546-0091 USA; 5 Departamento de Microbiología Molecular, Instituto de Biotecnología, Universidad Nacional Autónoma de México, Apdo. Postal 510-3, Cuernavaca, 62250 Morelos México

**Keywords:** *Bacillus thuringiensis*, *Plutella xylostella*, CRISPR/Cas9, ABC transporters, GPI-anchored proteins, Receptor redundancy, Toxin action

## Abstract

**Background:**

Biopesticides and transgenic crops based on *Bacillus thuringiensis* (Bt) toxins are extensively used to control insect pests, but the rapid evolution of insect resistance seriously threatens their effectiveness. Bt resistance is often polygenic and complex. Mutations that confer resistance occur in midgut proteins that act as cell surface receptors for the toxin, and it is thought they facilitate its assembly as a membrane-damaging pore. However, the mechanistic details of the action of Bt toxins remain controversial.

**Results:**

We have examined the contribution of two paralogous ABC transporters and two aminopeptidases N to Bt Cry1Ac toxicity in the diamondback moth, *Plutella xylostella*, using CRISPR/Cas9 to generate a series of homozygous polygenic knockout strains. A double-gene knockout strain, in which the two paralogous ABC transporters ABCC2 and ABCC3 were deleted, exhibited 4482-fold resistance to Cry1A toxin, significantly greater than that previously reported for single-gene knockouts and confirming the mutual functional redundancy of these ABC transporters in acting as toxin receptors in *P. xylostella*. A double-gene knockout strain in which APN1 and APN3a were deleted exhibited 1425-fold resistance to Cry1Ac toxin, providing the most direct evidence to date for these APN proteins acting as Cry1Ac toxin receptors, while also indicating their functional redundancy. Genetic crosses of the two double-gene knockouts yielded a hybrid strain in which all four receptor genes were deleted and this resulted in a > 34,000-fold resistance, indicating that while both types of receptor need to be present for the toxin to be fully effective, there is a level of functional redundancy between them. The highly resistant quadruple knockout strain was less fit than wild-type moths, but no fitness cost was detected in the double knockout strains.

**Conclusion:**

Our results provide direct evidence that APN1 and APN3a are important for Cry1Ac toxicity. They support our overarching hypothesis of a versatile mode of action of Bt toxins, which can compensate for the absence of individual receptors, and are consistent with an interplay among diverse midgut receptors in the toxins’ mechanism of action in a super pest.

**Supplementary Information:**

The online version contains supplementary material available at 10.1186/s12915-022-01226-1.

## Background

The entomopathogenic bacterium *Bacillus thuringiensis* (Bt) produces different types of insecticidal crystal proteins which are used to control insect pests both in sprayable formulations and transgenic crops [1]. Bt-based biotechnology products are the most successful alternatives to chemical pesticides for insect pest control [2, 3]. However, the economic, environmental, and social benefits of these Bt products are threatened by the evolution of insect resistance [4, 5]. To date, field-evolved Bt resistance has been observed in at least nine different lepidopteran and one coleopteran species [6]. Although a range of countermeasures have been introduced to prevent the development of resistance in diverse insect populations [7–11], an in-depth unraveling of the molecular mechanisms underlying Bt mechanism of action and resistance is particularly important for the improvement of Bt technology and its sustainability [12].

Bt Cry toxins are toxic upon ingestion by the host insect and target the midgut epithelium, the interactions of Cry toxins with their midgut-specific receptors trigger toxin oligomerization and pore formation [13, 14]. The best established Bt resistance mechanism in insects is via alterations of these midgut receptors such as cadherin (CAD), aminopeptidases-N (APNs), alkaline phosphatases (ALPs), and ABC transporters, that interrupt toxin binding [15, 16]. Among these receptors, structural mutations or expression alterations of ABC transporters (especially ABCC2) have been widely reported to be associated with high levels of resistance in lepidopteran insects [17–28]. In addition, as the first identified functional receptors of Bt Cry toxins, GPI-anchored APN proteins also play a crucial role [27, 29–32]. The alteration of a single receptor gene can however affect only one pathway of toxicity for Cry toxins [33]. Numerous instances of pest resistance to chemical insecticides are polygenic, and the eventual levels of resistance are decided by the interactions of different resistance alleles [34, 35]. Previous studies suggested that the interactions of ABC transporters with cadherin could enhance cell toxicity, and co-operation between these two receptors was demonstrated by in vitro ectopic expression in different cell lines [36–39].

With the emergence of complete whole genome information for insects, the clustered regularly interspaced short palindromic repeats/CRISPR-associated protein 9 (CRISPR/Cas9) system has been used to explore gene function. The creation of multiple or large fragment gene deletions by the CRISPR method makes it possible to decipher the relationship between functional genes in host insects [40–42]. Besides the easily achieved single-gene knockouts [25, 27], double-gene knockout of paralogous midgut receptor genes with the CRISPR/Cas9 system has recently been conducted to confirm their in vivo functions with respect to Bt toxin receptors [43–45]. The molecular mechanism underlying insect resistance to Bt toxins is often perplexing and multifaceted, with the resistance trait associated with different types of midgut receptors [46]. Resistance to Cry1A toxins in the strains of *Plutella xylostella* that we are studying is associated with the down-regulation of multiple receptors including ABCC2, ABCC3, ABCB1, ABCG1, APN1, APN3a and ALP [25, 27] but is not associated with any mutation in, or change in expression of, cadherin [47, 48].

In this study, CRISPR/Cas9-mediated double knockouts of two pairs of the same types of midgut receptor genes (*PxAPN1*/*PxAPN3a* and *PxABCC2/PxABCC3*) resulted in small and moderate levels of resistance enhancement, respectively, when compared to knocking out the individual receptors [25, 27]. However, when all four receptors were knocked out, an extremely high level of resistance was observed. This result provides a multiple pathway model for understanding how different types of midgut receptors are involved and interplay in Bt pathogenicity and resistance.

## Results

### Construction of double knockout strains by CRISPR/Cas9

**Fig. 1:**
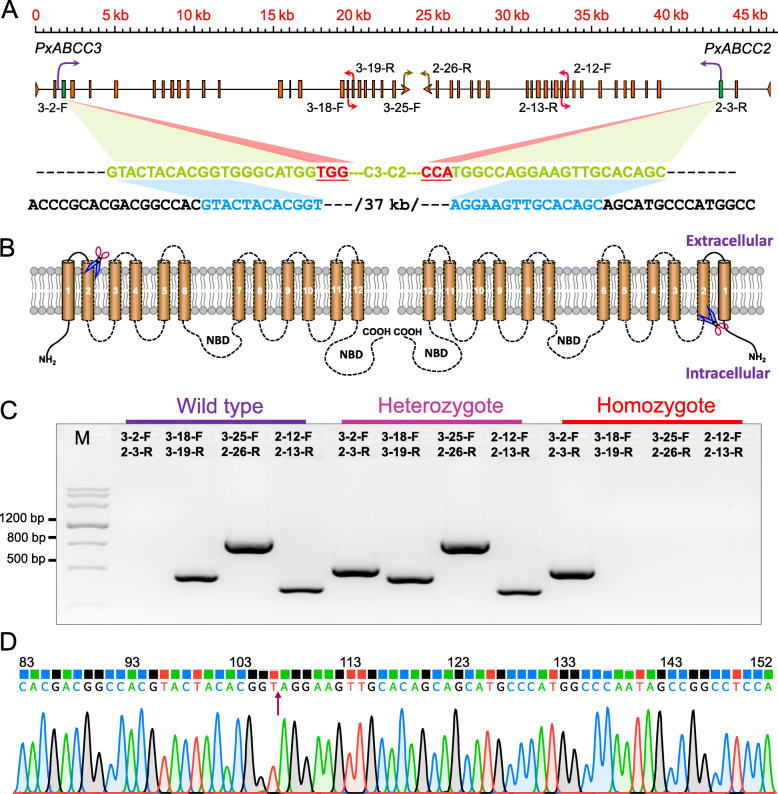
CRISPR/Cas9-mediated double gene mutation of *PxABCC2* and *PxABCC3* in *P. xylostella*. **a** The contiguous genomic structure of *PxABCC2* and *PxABCC3*. Exons are shown as boxes except for the first and last exons which are represented by triangles and arrows respectively. The sgRNA target sequences of *PxABCC2* and *PxABCC3* are highlighted by green text, the PAM sites are highlighted in red and underlined. The remaining sequences after cleavage are shown in blue. **b** Protein structures of PxABCC2 (right) and PxABCC3 (left), as well as the cleavage position following editing. **c** Genotyping of individual *P. xylostella* for deletion of *PxABCC2* and *PxABCC3* according to the banding profile of PCR products from the four primer pairs. **d** Representative chromatograms of direct sequencing of PCR products with the primer pair 3-2-F/2-3-R, the arrow indicates the junction of *PxABCC2* and *PxABCC3* after editing. In the nomenclature of these primers, the first number denotes the gene, the second number indicates the exon/intron. M: DNA marker III

**Fig. 2:**
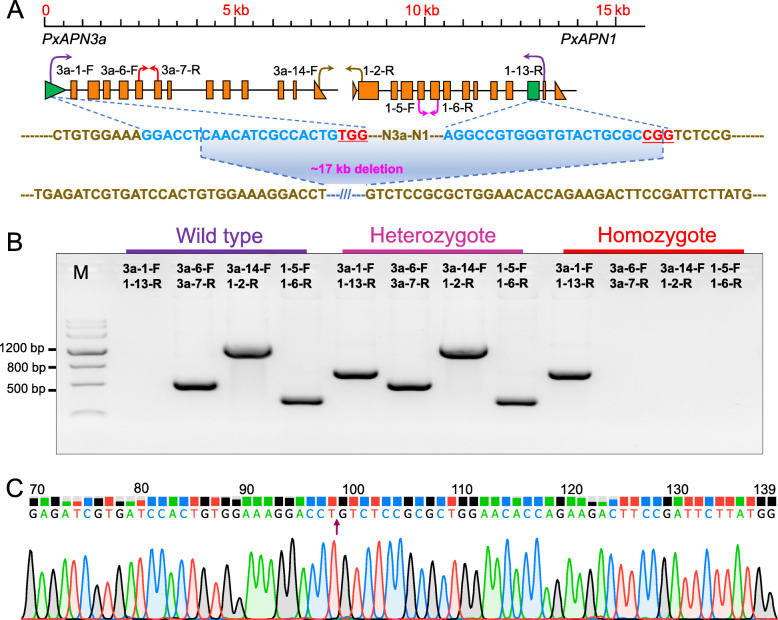
CRISPR/Cas9-based double gene knockout of *PxAPN1* and *PxAPN3a* in *P. xylostella*. **a** The genomic structure of *PxAPN1* and *PxAPN3a*, the sgRNA sequences are highlighted in blue text and the PAM sequences by underlined red text. The disrupted sequences are shown in the gray-blue dashed box. **b** Genotype detection of double gene mutation in *PxAPN1* and *PxAPN3a* locus according to the banding profile of PCR products from the four primer pairs. **c** Representative chromatograms of direct sequencing of PCR products with the primer pair 3a-1-F/1-13-R. The arrow indicates the junction of *PxAPN1* and *PxAPN3a* genes after deletion. M: DNA marker III

Based on the fact that the *PxABCC2* and *PxABCC3* receptor genes are adjacent on the *P. xylostella* genome, as are the *PxAPN1* and *PxAPN3a* receptor genes, a dual sgRNA CRISPR/Cas9 approach was used to knockout each pair of genes. Fresh eggs from the susceptible DBM1Ac-S strain were collected and co-injected with a mixture of Cas9 protein and two sgRNAs targeting either *PxABCC3* and *PxABCC2* (group 1) or *PxAPN3a* and *PxAPN1* genes (group 2) to obtain G0 progeny (Figs. 1a, b and 2a).

Subsequently, single-pair reciprocal crosses between G0 and DBM1Ac-S were performed to generate G1 progeny (Table 1). Single pairs which produced fertile progeny were screened and ten second-instar *P. xylostella* larvae from each of these pairs were pooled to extract gDNA samples. PCR amplification with primer pair 3-2-F/2-3-R exhibited a 516-bp fragment (indicating a successful deletion) in only a single pair in group 1 (Fig. 1c). This PCR product was then directly sequenced using 3-2-F as primer. The sequencing chromatogram revealed successful mutagenesis of *PxABCC2* and *PxABCC3* genes (Fig. 1d) and the remaining larvae were reared to pupation. In group 2, PCR amplification with primer pair 3a-1-F/1-13-R revealed a 716 bp fragment in two single pairs (Fig. 2b), PCR products were directly sequenced in these two pairs using 3a-1-F to confirm the mutagenesis within the *PxAPN3a* and *PxAPN1* locus (Fig. 2c). The pair group with the most larvae was chosen for further rearing. A nondestructive method for extracting the gDNA samples was used to screen pupae for the presence of the mutation (Figs. 1d and 2c) and heterozygotes containing the mutation were sib-mated to produce G2 progeny. Finally, homozygous individuals identified by PCR and DNA sequencing were further sib-crossed to establish two stable homozygous mutant strains designated C2-3KO (ABC transporter) and N1-3aKO (APN) (Additional file 1: Fig. S1).

**Table 1:** Mutagenesis of multiple midgut receptors mediated by CRISPR/Cas9 in *P. xylostella*

Strains	G0				G1		G2	
	Eggs	Hatched (%)^#^	Adults (%)^&^	Reciprocal crosses*	Fertile progeny (%)^†^	Heterozygous (%)^‡^	Pupae	Homozygous (%)^¶^
C2-3KO	450	152/450 (34)	68/152 (45)	25 × 25	20/50 (40)	30/70 (43)	100	18/100 (18)
N1-3aKO	468	173/468 (37)	75/173 (43)	25 × 25	22/50 (44)	35/80 (44)	96	24/96 (25)

^#^Among the injected eggs, approximately 34% (152/450) and 37% (173/468) hatched to larvae in C2-3KO and N1-3aKO groups

^&^Among the hatched larval, about 45% (68/152) and 43% (75/173) of the larvae developed into adults in G0

*25 single-pair reciprocal crosses between the G0 progeny and the susceptible DBM1Ac-S were performed to produce G1 offspring

^†^Among the 50 single pairs, 20/22 single pairs generate fertile progeny and one or two of the 20/22 single pairs showed mutagenesis in *PxABCC3* and *PxABCC2* or *PxAPN3a* and *PxAPN1* locus by the specific PCR amplification

^‡^The other larvae from the single pair group with the positive mutation were reared to pupation, the gDNA samples from 70/80 exuviates of the fourth-instar larvae in C2-3KO/N1-3aKO group were used for nondestructive detection, and 30/35 of them were heterozygotes

^¶^18/24 homozygous individuals were determined by direct sequencing using the gDNA samples from 100/96 exuviates of the final fourth-instar larvae in C2-3KO or N1-3aKO group

### Combinational inactivation of ABC transporters and GPI-anchored proteins

To produce a combined mutant strain containing both of the above deletions in the ABC transporter and APN genes, 30 single-pair reciprocal crosses were performed between C2-3KO and N1-3aKO (Additional file 1: Fig. S2). Among the 30 single pairs, 26 single pairs produced fertile progeny, a total of 350 individuals obtained in G1 were mass crossed to generate G2 offspring. A total of 450 third-instar larvae were screened with a diagnostic dose (10 mg/L) of Cry1Ac protoxin, which would be expected to kill all susceptible larvae. Surviving individuals were reared to pupation, and their genotypes were identified by PCR. Ultimately, 35 individuals (16 females and 19 males) with both 516 bp and 716 bp PCR bands were selected (Additional file 1: Fig. S2) and sib-crossed again to create another stable homozygous mutant strain named C-NKO with *PxABCC2*, *PxABCC3*, *PxAPN1* and *PxAPN3a* deleted in G3.

### Cross-resistance of polygenic knockout strains to Cry1A toxins

Our previous studies have shown that knocking out the *PxABCC2*, *PxABCC3*, *PxAPN1*, and *PxAPN3a* genes in *P. xylostella* caused 724, 413, 463, and 346-fold resistance to Cry1Ac toxin, respectively [25, 27]. Bioassays were subsequently performed to test the susceptibility changes to Cry1A protoxins among the newly-built strains in this study with the susceptible DBM1Ac-S used as a control (Table 2). The results indicated that the LC_50_ values of the double knockout strains C2-3KO and N1-3aKO to Cry1Ac protoxin were approximately 4482 and 1425-fold higher than that of the susceptible DBM1Ac-S strain, respectively. The quadruple knockout strain C-NKO showed > 34,000-fold resistance to Bt Cry1Ac protoxin. The three polygenic knockout strains also manifested high levels of cross-resistance to Cry1Ab protoxin (C2-3KO: 591-fold; N1-3aKO: 132-fold; C-NKO: > 4200-fold) but no cross-resistance to Cry1Aa protoxin was detected (C2-3KO: 1.2-fold; N1-3aKO: 1.3-fold; C-NKO: 1.5-fold). These data demonstrated the involvement of *PxABCC2* and *PxABCC3*, as well as *PxAPN1* and *PxAPN3a* genes in the mechanism of action of Cry1Ac and Cry1Ab in *P. xylostella*.

**Table 2:** Resistance to Cry1 toxin in larvae from the susceptible (DBM1Ac-S) and polygenic knockout strains

Toxins	Strains	N*	LC_50_ (95% CL)^†^	Slope ± SE	*χ*^2^(df)^‡^	RR^§^
Cry1Ac	DBM1Ac-S	210	0.73 (0.56–0.93)	2.02 ± 0.24	3.14(5)	1.0
	C2-3KO	210	3271.57 (2604.98–4198.52)	2.34 ± 0.28	4.80(5)	4482
	N1-3aKO	210	1040.23 (784.13–1463.05)	1.74 ± 0.23	1.58(5)	1425
	C-NKO	210	> 25,000^¶^	—	—	> 34,247
Cry1Ab	DBM1Ac-S	210	0.70 (0.54–0.89)	2.18 ± 0.26	3.31(5)	1.0
	C2-3KO	210	413.77 (325.22–543.91)	2.19 ± 0.28	0.60(5)	591
	N1-3aKO	210	92.37 (71.44–120.50)	1.89 ± 0.22	1.20(5)	132
	C-NKO	210	> 3000^¶^	—	—	> 4286
Cry1Aa	DBM1Ac-S	210	0.72 (0.48–1.03)	2.07 ± 0.24	6.16(5)	1.0
	C2-3KO	210	0.87 (0.66–1.12)	1.93 ± 0.22	3.16(5)	1.2
	N1-3aKO	210	0.93 (0.72–1.19)	2.04 ± 0.23	2.76(5)	1.3
	C-NKO	210	1.05 (0.81–1.36)	1.93 ± 0.22	3.46(5)	1.5

*Number of larvae tested (larvae of the control group not included)

^†^Concentration of Cry1Ac toxin (mg/L) killing 50% of larvae and its 95% confidence limits (CL)

^‡^The value of chi-square and degrees of freedom (df) were calculated by Polo Plus 2.0

^§^RR: Resistance ratio (RR) calculated by LC_50_ of the polygenic knockout strains divided by LC_50_ of the susceptible DBM1Ac-S strain

^¶^Both Cry1Ac and Cry1Ab toxins produced < 15% mortality in larvae from the C-NKO strain when examined with the Cry1Ac concentration of 2.5× 10^4^ mg/L and 3× 10^3^ mg/L

### Inheritance of resistance to Cry1Ac in the engineered strains

**Fig. 3:**
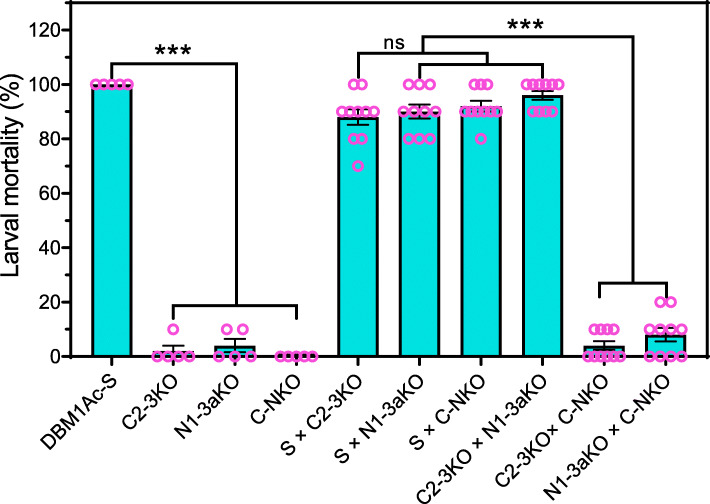
Interstrain allelic complementation test with a diagnostic dose of Cry1Ac protoxin. F1 progeny were generated by six interstrain crosses between DBM1Ac-S, C2-3KO, N1-3aKO, and C-NKO. The mortality of the resulting strains (50 larvae from each strain) and their F1 progeny (100 larvae from each F1 offspring) was examined with a diagnostic dose (10 mg/L) of Cry1Ac protoxin, which should kill 100% heterozygous F1 larvae. Significant differences in larval mortality were analyzed by one-way ANOVA. The *p* values from Holm-Sidak’s test, *p < 0.05, **p < 0.01, ***p < 0.001, ns, not significant

To analyze the inheritance of Cry1Ac resistance in the polygenic knockout strains, F1 progeny were mass crossed between pairs of strains from DBM1Ac-S, C2-3KO, N1-3aKO, and C-NKO, and resulting larvae were screened with 10 mg/L Cry1Ac protoxin to remove all susceptible individuals. As expected, the mortality was 100% for the susceptible DBM1Ac-S strain while the F1 progeny of the knockout strains had a high survival rate (96-100%) (Additional file 1: Table S2). F1 progeny from crosses between either double mutant strain C2-3KO or N1-3aKO or the quadruple knockout C-NKO strain and DBM1Ac-S showed relatively low survival rates (8-12%) as did progeny from the C2-3KO vs N1-3aKO cross (4%). In contrast, progeny from crosses between C-NKO and the two double mutant strains showed high survival rates (92–96%) (Fig. 3). These results confirm that the C2-3KO and N1-3aKO strains share different, essentially recessive, resistance loci, and that allelic complementation restored susceptibility to Cry1Ac protoxin and showed a wild-type phenotype.

### Genetic linkage of polygenic mutations with Cry1Ac resistance

**Fig. 4:**
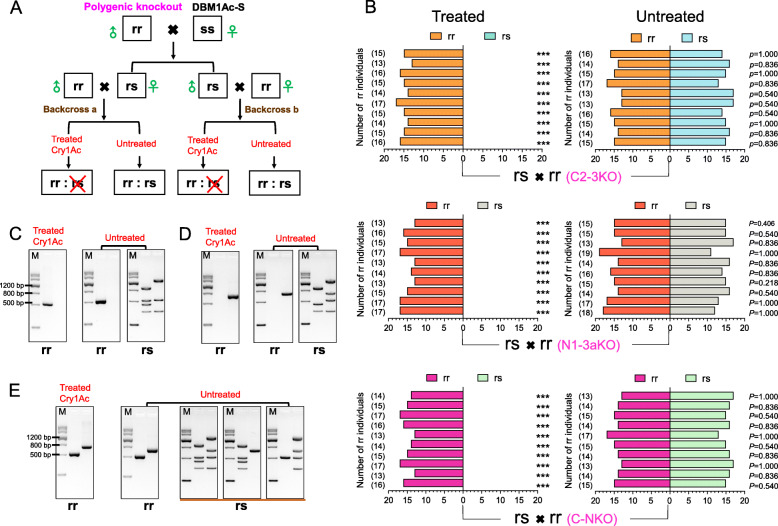
Genetic linkage analysis of Cry1Ac resistance in the polygenic knockout strains of *P. xylostella*. **a** The crossing strategy for analysis of the linkage between polygenic knockout and Cry1Ac resistance. **b** To examine the genetic linkage between multiple resistance alleles and Cry1Ac resistance, 10 backcross families from the single-pair cross between C2-3KO (rr) and their F1 progeny (rs, C2-3KO×DBM1Ac-S) (top pictures), N1-3aKO (rr), and their F1 progeny (rs, N1-3aKO×DBM1Ac-S) (middle pictures), as well as C-NKO (rr) and their F1 progeny (rs, C-NKO×DBM1Ac-S) (bottom pictures) were used. Each picture contains 10 backcross families from bottom to top, the first lines 1-5 represent backcross a, and the following lines 6-10 showed backcross b. Bioassays were performed on 30 larvae from each backcross group, and a total of 300 larvae were treated with the diagnostic doses of Cry1Ac protoxin, another 300 untreated larvae were also collected as controls. The genotypes of individuals that were or were not treated with Cry1Ac were detected by the band size of the PCR products with the four primer pairs in *PxABCC2* and *PxABCC3* or *PxAPN1* and *PxAPN3a* locus (shown in Additional file 1: Table S1). The number of individuals with the genotype of rr is listed on the left side of these figures, and the significant difference of genotype ratio in Cry1Ac treated or not backcross groups were calculated by the Fisher’s exact test, and the *p* values are shown on the right of figures, *p < 0.05, **p < 0.01, ***p < 0.001. **c**–**e** Genotype detection of F2 backcross families from crossing the polygenic knockout strains (C2-3KO, N1-3aKO, and C-NKO, respectively) and their corresponding F1 progeny. The gDNA samples of all the individuals from F2 backcross families, that were or were not exposed to Cry1Ac toxin, were extracted for PCR amplification using the four primer pairs in *PxABCC2*/*PxABCC3* or *PxAPN1*/*PxAPN3a* loci (shown in Figs. 1c and 2b). PCR products were purified and resolved by 1.5% agarose gel electrophoresis

We further carried out genetic linkage analysis to test the cosegregation of different multiple gene inactivations with resistance to Cry1Ac in our polygenic knockout strains. Single-pair crosses between a male from the polygenic knockout strains (C2-3KO, N1-3aKO, or C-NKO) and a female from the susceptible DBM1Ac-S were performed to generate F1 progeny. After five reciprocal crosses between the F1 progeny and the respective polygenic knockout moths were performed to produce ten F2 backcross families (named backcross a and backcross b, as labeled in Fig. 4a), individuals were or were not treated with a diagnostic dose of Cry1Ac protoxin (Fig. 4a, b). The genotypes of individuals were assessed by PCR analysis (Fig. 4c–e).

Among 300 untreated progeny from the C2-3KO cross, almost half of the individuals were heterozygous (152) and the rest (148) were homozygous for the *PxABCC2* and *PxABCC3* mutations (Fig. 4c). The genotype ratio (rr:rs) between the number of moths in backcross a and backcross b were 73:77 and 75:75, respectively, which was consistent with the 1:1 random assortment ratio (*p* > 0.5 or *p* = 1.0, Fisher’s exact test) (Fig. 4b, top right). When treated with the diagnostic dose of Cry1Ac (20 mg/L), only homozygous individuals were detected and accounted for about 50% (51% for backcross a, 49% for backcross b), which followed the expected Mendelian inheritance of a recessive resistance trait (*p* < 0.001, Fisher’s exact test) (Fig. 4b, top left). A similar result was obtained in N1-3aKO with the genotype ratio (rr:rs) between the number of moths in backcross a and backcross b being 80:70 and 76:74 (*p* > 0.5 or *p* = 1.0, Fisher’s exact test) (Fig. 4b, middle right). Rearing the larvae of each backcross with the diagnostic dose of Cry1Ac (20 mg/L) caused approximately 50% mortality (51% for backcross a, 49% for backcross b) (*p* < 0.001, Fisher’s exact test) (Fig. 4b, middle left). These results suggest that the mutations of *PxABCC2*/*PxABCC3* or *PxAPN1/PxAPN3a* genes are closely linked with resistance to Cry1Ac in the C2-3KO and N1-3aKO strains, respectively.

For the C-NKO strain, the genotype ratio (rr:rs) between the number of moths in backcross a and backcross b were 71:79 and 73:77 (Fig. 4b, bottom right). Rearing the larvae of each backcross with the diagnostic dose of Cry1Ac (4000 mg/L) caused 50% mortality for both backcrosses a and b (*p* < 0.001, Fisher’s exact test). This further demonstrated that the combined mutations of ABC transporters and GPI-anchored proteins are also tightly linked to Cry1Ac resistance in the C-NKO strain.

### Fitness cost analysis

**Fig. 5:**
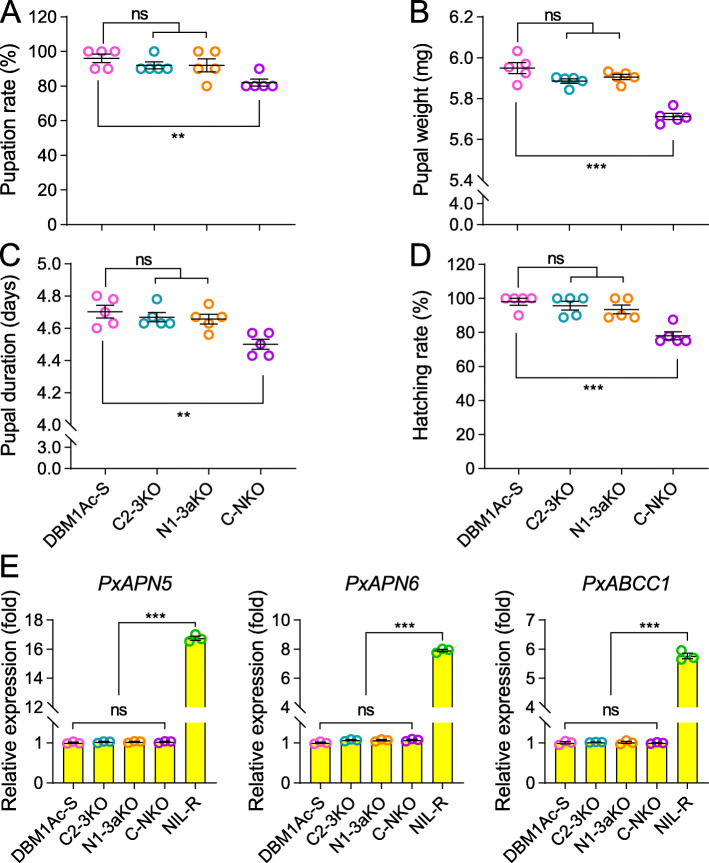
Evaluation of fitness costs in polygenic editing strains. A series of biological parameters were compared in the polygenic knockout strains (C2-3KO, N1-3aKO, and C-NKO) with the susceptible DBM1Ac-S strain. **a** Pupation percentage. **b** Pupal weight. **c** Pupal duration. **d** Hatching rate. **e** The expression level of non-receptor genes in the DBM1Ac-S, C2-3KO, N1-3aKO, C-NKO, and NIL-R strains. Data are presented as mean value ± SEM, *n* = 5 biologically independent samples with ten larvae per replicate for a–d, and *n* = 3 biologically independent samples for e, *p < 0.05, **p < 0.01, ***p < 0.001, ns, not significant. One-way ANOVA with Holm-Sidak’s test was used for comparison

Fitness costs related to life-history traits are frequently associated with insect resistance to Bt pathogens thereby influencing the evolution of resistance [49]. To confirm whether individuals of the different knockout strains had growth penalties, a series of biological parameters including pupation rate, pupal weight, pupal duration, and hatching percentage were assessed. We observed that the double mutant C2-3KO and N1-3aKO strains had no significant differences when compared to the susceptible DBM1Ac-S strain, whereas the quadruple knockout strain C-NKO displayed some fitness disadvantages in all of the measured parameters (Fig. 5a–d). Previously, we had found no fitness costs associated with Cry1Ac resistance in a strain in which down-regulation of the functional receptors PxABCC2/3 and PxAPN1/3a was accompanied by the up-regulation of the non-receptor paralogs PxABCC1 and PxAPN5/6. We speculated that this compensation mechanism minimized fitness costs while maintaining a high level of resistance [22, 27, 50]. Given the fitness costs associated with the quadruple knockout C-NKO (but not the two double knockout strains) we investigated the expression of the non-receptor paralogs in these three strains. We observed no differences in expression of *PxAPN5/6 or PxABCC1* in any of the knockout strains compared to the parent (Fig. 5e). This observation was consistent with fitness costs in the quadruple knockout strain being associated with the non-compensated loss of the functional receptors, although could not explain the lack of fitness costs associated with the double knockouts.

## Discussion

**Fig. 6:**
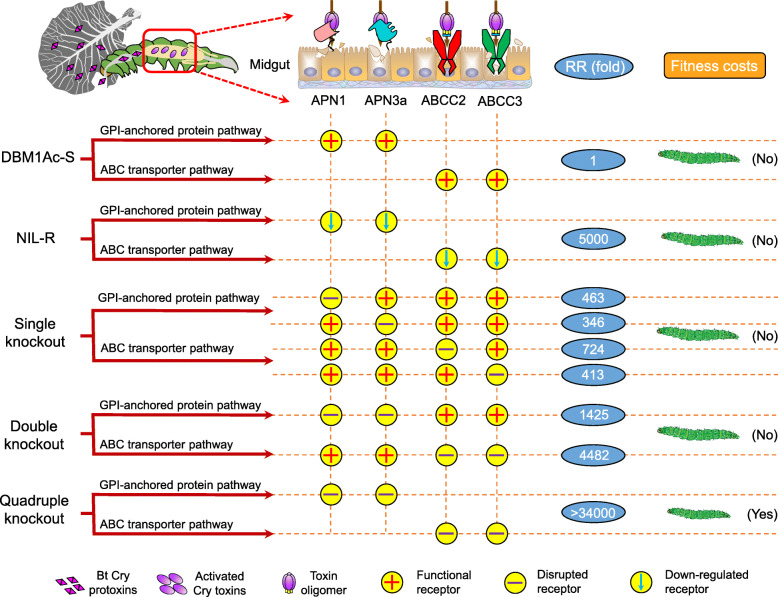
Diagramatic representation of the response of *P. xylostella* larvae from multiple strains after treatment with Cry1Ac toxin. The dashed lines to which the arrow points represent the pathways with the corresponding receptors changed. Different *P. xylostella* strains: susceptible DBM1Ac-S strain; near-isogenic Cry1Ac-resistant NIL-R strain; CRISPR/Cas9-mediated single mutant strain with *PxAPN1*/*PxAPN3a*/*PxABCC2*/*PxABCC3* deleted, respectively; CRISPR/Cas9-mediated double mutant strain with *PxAPN1*/*PxAPN3a* or *PxABCC2*/*PxABCC3* deleted; the hybrid strain with both deletions. RR: Resistance ratio

The interactions between insect midgut receptors and the Bt pesticidal proteins influence both the pathogenicity of the bacterium and the ability of the host to evolve resistance. Interactions between resistance alleles are known to play a vital role in the evolution or maintenance of polygenic insecticide resistance in the field [34]. Resistance alleles based on mutations to midgut receptors are well known to affect the pathogenicity of Bt and more recently reports of interactions between these alleles have been noted. An interaction between ABCC2 and cadherin BtR175 in Bt Cry1Ab resistance was proposed in *Bombyx mori* [37] and similar results were obtained with Cry1Ac resistance in *Heliothis virescens* and *Trichoplusia ni* [17, 33, 36]. ABCC2 cooperated with its paralog ABCC3 triggering > 15,000 and > 8000-fold resistance to Bt Cry1Ac toxin in *Helicoverpa armigera* and *P. xylostella*, respectively, when both were knocked out, however, mutations in either of these single genes had little or no effect [43, 44]. Other reports observed levels of resistance when either ABCC2 or ABCC3 were knocked out individually [25, 28] but much higher levels were reported for the double knockouts. In this study, a CRISPR/Cas9-mediated double knockout of *ABCC2* and *ABCC3* in *P. xylostella* resulted in a significantly greater level of resistance than was previously observed with individual knockouts [25, 27]. Although the individual resistance ratios of the single and double knockouts varied between studies [43, 44], there was a consistent pattern of the double knockout resulting in a greatly increased resistance (Fig. 6), indicating functional redundancy between the two ABCC receptors. In addition, we showed that knocking down the two APN receptors also resulted in a significant increase in resistance to Cry1Ac, suggesting that these APN receptors have a redundant function (Fig. 6). The level of Cry1Ac resistance in the quadruple knockout strain was so high that it was impossible to calculate an LC_50_ value.

A commonly cited model for Bt toxin mechanism of action is the “sequential binding” model in which toxin monomers are proposed to the first bind to a membrane-anchored receptor increasing the concentration of toxins at the membrane surface. Subsequently, the toxin interacts with a second receptor which induces oligomerization of the protein following a proteolytic cleavage. Insertion of the oligomer into the membrane to form a pore may be facilitated by it further binding to a lipid raft-associated protein [13, 15]. In a model in which a defined and obligatory sequential movement of the toxin between different receptors exists, it logically follows that blocking any one of the steps would lead to similar levels of resistance. For example, if movement between an APN and an ABCC receptor is a required step in the mechanism, then removing either the APN receptor(s) or the ABCC receptor(s) would lead to the same level of resistance. However, if redundant receptors with the same (receptor) function exist in the same insect, this would require multiple receptors to be knocked out in order to have high levels of resistance. In *P. xylostella* any redundancy between APN and ABCC proteins is most likely to exist in one of two ways. Firstly the two APN proteins substitute for each other at one stage of the sequential pathway and the two ABCC proteins at another stage. In such a scenario knocking out both the APNs, or both the ABCC proteins, would block the pathway and give similarly high levels of resistance. Secondly, all four proteins could independently act as receptors in a non-sequential pathway in which case all four would have to be knocked out to give high levels of resistance. The data presented here support the latter idea that there is full redundancy between the *PxAPN1*/*3a* and *PxABCC2/3* receptors. There is good evidence from ectopic expression studies that APN and ABCC proteins are alone capable of facilitating Cry protein toxicity [27, 36, 51–54]. Thus, although our data suggest that these four receptors are functionally redundant and independent, they are also consistent with the possibility of some cooperation between them to enhance the activity of the Cry protein.

There is convincing evidence in the literature for synergistic interactions between different receptors within the Bt toxin mechanism of action. For example, Bretschneider et al. [36] demonstrated that ectopic expression of the *H. virescens* ABCC2 receptor in a non-susceptible cell line could render that line susceptible to Cry1Ab. Ectopic expression of the cadherin receptor from the same insect resulted in little or no change in susceptibility, yet when both receptors were expressed in the same cell, the susceptibility was much greater than the additive effect of the two alone. Further studies using ABCC2 and cadherin receptors from *H. armigera*, *Spodoptera litura*, and *Spodoptera exigua* expressed in cell lines also observed synergistic reactions [55, 56] as did studies in which the receptors were expressed in *Xenopus*oocytes [39] or in *Drosophila melanogaster* [57]. Work involving both naturally occurring and artificially created mutations in *H. armigera* ABCC2 and cadherin also showed evidence of synergistic interaction [58]. The mechanistic detail of this synergistic effect remains unclear although Heckel [59] has outlined a number of cis- and trans-acting models. A common theme of these synergism models is that some receptors may be better at inducing oligomer formation whereas others may be better at facilitating insertion into the membrane. ABCC2 has been shown to be capable of enabling both of these processes, inducing oligomer formation and its insertion into the membrane [60], the overall effect though could be enhanced via cooperation with a second receptor such as an APN.

The pyramid strategy of transgenic Bt crops effectively controls insect pests by producing two or more toxins that kill the same pest [9]. However, the method is often ineffective in delaying or overcoming resistance because resistance to one toxin is often accompanied by cross-resistance to others [61]. In our laboratory bioassays of *P. xylostella*, we discovered that the knockout strains C2-3KO, N1-3aKO, and C-NKO showed resistance to Cry1Ac and Cry1Ab but no obvious cross-resistance to Cry1Aa (Table 2). Various models describing multiple Cry protein binding sites in *P. xylostella* have been proposed [62], although these were derived based on resistant strains to which Cry1Aa bound but was not toxic. Although some resistant *P. xylostella* strains show cross-resistance to Cry1Aa, resistance ratios to this toxin are often less than those for Cry1Ab or Cry1Ac [63, 64]. Our data are consistent with the primary functional receptor for Cry1Aa being something other than APN1/3a or ABCC2/3, perhaps ALP which is known to be associated with resistance to Cry1Aa expressing Bt strains in other *P. xylostella* strains [22, 27].

In nature, the evolutionary outcome of insect resistance to insecticides is determined by the fitness costs of each resistance allele, and the overall fitness disadvantages are traded off against the advantages of the resistance phenotype [34, 65, 66]. Since many Cry toxin receptors are physiologically important proteins in the insect, resistance-inducing mutations or loss of expression can alter digestive processes. However, the up-regulation of paralogs with no receptor function and transcriptional plasticity could compensate for the functional impairment [67, 68]. Our previous studies have shown that the MAPK signaling pathway can *trans*-regulate the differential expression of multiple midgut genes, including *PxABCC1-3* and four *PxAPN* genes (*PxAPN1*, *PxAPN3a*, *PxAPN5*, and *PxAPN6*), thereby countering the virulence effect of Bt toxin in *P. xylostella* without occurring significance fitness costs [22, 27, 50]. Among them, the up-regulation of *PxABCC1*, *PxAPN5*, and *PxAPN6* were not associated with Cry1Ac resistance. This up-regulation is speculated to make up for the loss of function of *PxABCC2*, *PxABCC3*, *PxAPN1*, and *PxAPN3a* and diminish the fitness cost of Cry1Ac resistance. A similar compensation mechanism of *TnAPN1* and *TnAPN6* was reported in Cry1Ac-resistance *T. ni* [30]. In this work, we found that the double mutant C2-3KO and N1-3aKO strains exhibited no statistically significant differences in the measured developmental or physiological features, although such a difference was observed in the quadruple knockout strain C-NKO (Fig. 6). One possible explanation for the lack of large fitness costs in the double knockouts is that the insect compensated for the loss of the receptor proteins through the upregulation of receptor paralogs, perhaps via hormonal signaling pathways that have previously been associated with resistance and insect fitness [69, 70]. However, when we measured the levels of the non-receptor paralogs *PxAPN5/6* and *PxABCC1* we found no change in any of the knockout strains. This would indicate that no simple feedback system exists to compensate for the loss of those midgut proteins. It remains unclear why the two double knockout strains had little effect on fitness whereas the quadruple one did, although it could just be that individual knockouts incur small fitness penalties that are amplified when combined. Related work recently reported for *H. armigera* [58] found that in a strain where resistance had been linked to a mutation in *HaABCC2*, expression of *HaABCC3* was also down-regulated, whereas this was not the case in an artificially constructed *HaABCC2* knockout. This result and our previous characterization of resistant *P. xylostella* strains indicate that, unlike lab-induced gene knockouts, naturally evolved resistant strains often present secondary effects that both increase the level of resistance while mitigating the physiological load on the insect.

## Conclusion

In summary, our data support the hypothesis that functional redundancy goes beyond ABC transporters to include other, more diverse, midgut receptors. Specifically, knockouts of individuals or pairs of a suite of Bt receptors, including APN1, APN3a, ABCC2, and ABCC3, result in some degree of resistance, while high levels of resistance are only seen when all four proteins are eliminated. Previous studies [25, 27, 57] have demonstrated that individual members of this group can act as receptors in their own right and so it appears that there is no requirement for multiple receptors to be present. Further research is warranted to address whether the effect of multiple receptors on susceptibility is purely additive or if there are synergistic interactions. The functional redundancy that we have identified among diverse midgut receptors not only supports our overarching hypothesis of a compensatory mode of action of Bt toxins in *P. xylostella*, but also provides a plausible explanation as to why the resistance mechanisms of this super pest involve a coordinated transcriptional programing of multiple receptor proteins [22, 27, 50].

## Methods

### Insect strains

The susceptible DBM1Ac-S strain and its near-isogenic Cry1Ac-resistant NIL-R strain of *P. xylostella* used in this study have been described in detail elsewhere [25, 71]. CRISPR/Cas9-mediated double mutant strains (C2-3KO and N1-3aKO) were constructed from the susceptible DBM1Ac-S strain by introducing 37 kb or 17 kb deletions into the *PxABCC2/PxABCC3* or *PxAPN1/PxAPN3a* gene loci resulting in loss of function of both encoded proteins. A hybrid strain (C-NKO) that contained mutations in all the above four genes was established by crossing the moths from C2-3KO and N1-3aKO strains. All *P. xylostella* strains were reared at 25 °C with 65% relative humidity (RH) and a photoperiod 16:8 (light: dark) on Jing Feng No. 1 cabbage (*Brassica oleracea* var. *capitata*), with a 10% honey/water solution for adults.

### In vitro synthesis of single guide RNA (sgRNA)

We designed four optimal sgRNAs targeting *PxABCC3*, *PxABCC2*, *PxAPN3a*, and *PxAPN1* genomic sequences (sgRNA1 targeting exon 3 of *PxABCC3*: GTACTACACGGTGGGCATGGTGG; sgRNA2 targeting exon 3 of *PxABCC2*: GCTGTGCAACTTCCTGGCCATGG; sgRNA3 targeting exon 1 of *PxAPN3a:* GGACCTCAACATCGCCACTGTGG; sgRNA4 targeting exon 12 of *PxAPN1:* AGGCCGTGGGTGTACTGCGCCGG, PAM site sequences were underlined) (Figs. 1a and 2a) according to the principle of 5′-N_20_NGG-3′ using the CRISPR RGEN tool Cas-Designer (http://www.rgenome.net/cas-designer/). The potential off-target effects of all the sgRNA target sequences were eliminated by searching in the *P. xylostella* genome database (DBM-DB, http://59.79.254.1/DBM/index.php), the GenBank database (https://www.ncbi.nlm.nih.gov/) and the CRISPR RGEN Cas-OFFinder tool (http://www.rgenome.net/cas-offinder/). The DNA template of sgRNAs was synthesized by a PCR-based fusion of an upstream oligonucleotide (T7 promotor sequences+sgRNA target sequences+GTTTTAGAGCTAGAAATAGC) and a downstream oligonucleotide encoding the reverse complement of the sgRNA sequences (Additional file 1: Table S1). High yields of sgRNAs were obtained by in vitro MEGAshortscript Transcription Kit (Ambion, Foster City, CA, USA) and purified by the MEGAclear Kit (Ambion, Foster City, CA, USA) following the given instructions.

### Preparation of sgRNA/Cas9 protein mixtures for microinjection

Freshly preblastoderm-stage eggs were laid on dry microscope slides (24 × 50 mm) precoated with fresh cabbage leaf juice to induce the spawning behavior of female adults. The *Streptococcus pyogenes* Cas9 protein was purchased from Thermo Fisher Scientific (Shanghai, China). About 1-nl mixture of sgRNAs and Cas9 protein were microinjected into individual eggs. The instruments and injection procedures were in line with our previous report [25]. For the double gene knockout, the two sgRNAs (sgRNA1 and 2 or sgRNA3 and 4) and Cas9 protein were simultaneously injected into individual eggs, and the final concentration of each sgRNA and Cas9 protein was both 100 ng/μl. The microinjection process was performed within 2 h, and the injected eggs were immediately incubated at 25 °C with 65% RH for hatching.

### Identification of double mutant moths mediated by CRISPR/Cas9

According to the arrangement of the *PxABCC3/PxABCC2* and *PxAPN3a*/*PxAPN1* genes in the DBM genome database, two primer pairs were designed to detect the mutagenesis of the two double-gene regions. 3-2-F is located in intron 2 of the *PxABCC3* gene as the forward primer and 2-3-R located in exon 3 of the *PxABCC2* gene as the reverse primer, a 516 bp genome DNA fragment was amplified with the primers 3-2-F/2-3-R if *PxABCC3* and *PxABCC2* genes were simultaneously deleted. 3a-1-F is located in exon 1 of the *PxAPN3a* gene as the forward primer, and 1-13-R located in exon 13 of the *PxAPN1* gene as a reverse primer, a 716 bp gDNA fragment was amplified with the primers 3a-12-F/1-13-R if the *PxAPN3a* and *PxAPN1* genes were deleted. To identify homozygous or heterozygous individuals, three other PCR primer pairs were designed between the two contiguous genes. For the identification of the *PxABCC3/ PxABCC2* knockout, the PCR primers 3-18-F/3-19-R are respectively located in exons 18 and 19 of the *PxABCC3* gene giving a PCR product of 441 bp, the PCR primers 2-12-F/2-13-R are located in exons 12 and 13 of the *PxABCC2* gene giving a PCR product of 334 bp, and the PCR primers 3-25-F/2-26-R are located at the junction of the *PxABCC3* and *PxABCC2* genes–the last exon 25 of the *PxABCC3* and the last exon 26 of the *PxABCC2* genes giving a PCR product of 891 bp. The mutations of the *PxAPN3a* and *PxAPN1* were similarly identified, PCR primers 3a-13-F/3a-14-R are located in exons 13 and exon 14 of *PxAPN3a* giving a 522 bp gDNA fragment, and PCR primers 1-10-F/1-11-R are located in exons 10 and 11 of *PxAPN1* gene giving a 332 bp PCR product fragment, an approximately 1381 bp PCR product between the *PxAPN3a* and *PxAPN1* genes was amplified using PCR primers 3a-14-F/1-2-R, which were respectively located in exon 14 of the *PxAPN3a* gene and exon 2 of the *PxAPN1* gene (Additional file 1: Table S1) Finally, the genotypes of the double mutant individuals (*PxABCC3*/*PxABCC2* and *PxAPN3a*/*PxAPN1*) were validated by the PCR profile of the resultant amplicons and DNA sequencing.

### Construction and identification of genetic hybridization strain

To generate a moth line with *PxABCC3*, *PxABCC2*, *PxAPN3a*, and *PxAPN1* gene mutations, a genetic cross strategy was employed. Taking into account that the *PxABCC3*/*PxABCC2* and *PxAPN3a*/*PxAPN1* genes in *P. xylostella* are orthologous to genes located in chromosome 15 or 9 of *B. mori*, respectively [18], we performed reciprocal crosses between lines C2-3KO (BBaa) and N1-3aKO (bbAA) that produce a heterozygous genotype (AaBb) (the aa represents the homozygous alleles for *PxABCC2* and *PxABCC3* deletions, and bb shows the homozygous alleles for *PxAPN1* and *PxAPN3a* mutations) (Additional file 1: Fig. S2). Subsequently, G1 heterozygous individuals were sib-crossed to produce G2 progeny and screened with a diagnostic dose (10 mg/L) of Cry1Ac protoxin which could kill all the susceptible larvae. The remaining surviving larvae were reared to pupation, and the gDNA samples of tiny exuviates were isolated from the final fourth-instar larvae to avoid damaging the pupae. Then, the genotype of individuals was detected by the profile of PCR amplicons using the corresponding four primer pairs between the *PxABCC3*/*PxABCC2* and *PxAPN3a*/*PxAPN1* loci (Additional file  1: Table S1). Finally, G2 individuals containing *PxABCC3*, *PxABCC2*, *PxAPN3a*, and *PxAPN1* mutations were selected and sib-mated to establishing a stable homozygous mutant strain designated C-NKO in G3.

### Toxins and bioassays

The Bt protoxins were extracted and purified from Btk strain HD-73 via the isoelectric point precipitation method as described previously [72]. All the Bt protoxins and trypsin-activated Bt toxins were quantified by Bradford’s method using bovine serum albumin (BSA) as a standard.

A 72 h leaf-disc-dip bioassay was performed to test the response of the DBM1Ac-S, C2-3KO, N1-3aKO, and C-NKO strains to three Bt Cry1A toxins (Cry1Aa, Cry1Ab, Cry1Ac) as reported elsewhere [71]. Ten third-instar larvae were respectively inoculated in seven gradient concentrations of each toxin, and each treatment was repeated three times. Larval mortality was recorded in each strain and the control mortality limit was set to 5%. After bioassays, the LC_50_ values (the concentration that killed 50% tested larval) and 95% CL (95% fiducial limits of LC_50_ values) values were calculated by Probit analysis using POLO Plus 2.0 software (LeOra Software, Berkeley, CA, USA). We examined the resistance ratio for each toxin as the LC_50_ values of each knockout strain divided by the LC_50_ of the susceptible DBM1Ac-S strain. The LC_50_ values of pairwise comparison were perceived as significantly different if their 95% CL did not overlap.

### Inheritance and allelic complementation analysis

To ascertain the inheritance of Cry1Ac resistance in both polygenic knockout strains, we used the same interstrain crossing strategy between two out of the DBM1Ac-S, C2-3KO, N1-3aKO, and C-NKO strains, the mortality of these four strains (50 larvae from each strain), and their F1 progeny (100 larvae from each F1 offspring) were examined with a diagnostic dose (10 mg/L) of Cry1Ac protoxin, which could kill 100% heterozygous F1 larvae. Then, the dominance degree (*h*) of Cry1Ac resistance was calculated with the formula reported elsewhere [73], with the value of *h* ranging from 0 (completely recessive) to 1 (completely dominant).

To confirm if the locus responsible for resistance to Bt Cry1Ac toxin changed among different strains, inter-strain allelic complementation tests were performed. Through bioassays, we examined the offspring of single-pair crossed between the DBM1Ac-S, C2-3KO, N1-3aKO, and C-NKO strains. If the moths from two resistant strains were crossed and the recessive resistance alleles were located in different loci, their progeny will exhibit susceptibility to Cry1Ac toxin, and if the recessive resistance alleles are located in the same locus, their progeny will be resistant to Cry1Ac toxin.

### Genetic linkage analysis

For genetic linkage analysis of double gene knockout strains (C2-3KO, N1-3aKO) and hybrid strain (C-NKO) with Cry1Ac resistance, a single-pair cross between a male from the knockout strains (C2-3KO, N1-3aKO, C-NKO) and a female from the susceptible DBM1Ac-S strain was performed to produce F1 progeny. Subsequently, five reciprocal crosses between an F1 progeny and a knockout moth were performed to generate ten F2 backcross families. A diagnostic dose of Cry1Ac protoxin that could kill all the F1 heterozygous larvae (20 mg/L for C2-3KO and N1-3aKO strains or 4000 mg/L for C-NKO strain) was used to confirm susceptibility by bioassay. For F2 progenies from each backcross family, 30 third-instar larvae were respectively reared on control cabbage or treated cabbage (20 mg/L or 4000 mg/L Cry1Ac protoxin smeared on the leaves) for 72 h. The mortality was recorded and the gDNA sample from each surviving larva was extracted for genotyping detection by PCR profile using the primers mentioned above for detecting the heterozygous or homozygous mutations in *PxABCC3*/*PxABCC2* or *PxAPN3a*/*PxAPN1* (Additional file 1: Table S1).

### Fitness cost analysis

We compared a series of physiological parameters of *P. xylostella* polygenic knockout strains to analyze the fitness cost caused by CRISPR/Cas9 and the genetic hybridization method. Biological parameters including pupation rate, pupation duration, pupal weight and hatching percentage were recorded, and larvae from the susceptible DBM1Ac-S were used as a control. Ten second-instar larvae from each strain were reared on fresh cabbage leaves without exposure to any Bt Cry toxin and the experiments were repeated in quintuplicate. The statistical significance difference in biological parameters between the control and polygenic knockout strains was determined by one-way ANOVA with Holm-Sidak’s test (overall significance level > 0.05).

### Quantitative PCR (qPCR) analysis

The transcript levels of non-receptor genes were quantified by real-time qPCR performed in a QuantStudio 3 Real-Time PCR System (Applied Biosystems). Gene-specific primers for *PxAPN5*, *PxAPN6*, and *PxABCC1* were used (Additional file 1: Table S1) in qPCR reactions with 2.5×SYBR Green MasterMix Kit (TIANGEN) as described in detail elsewhere [22, 27]. The qPCR program consisted of an original denaturation step for 6 min at 95 °C, subsequently, 40 cycles of denaturation for 30 s at 95 °C, annealing for 30 s at 53 °C for *PxAPN6*, 55 °C for *PxABCC1* and *PxAPN5*, followed by an extension for 35 s at 72 °C. Relative quantification was calculated by utilizing the 2^−ΔΔCt^ method and standardized to the ribosomal protein *L32* gene (GenBank accession no. AB180441). Three biological repetitions and four technical replicates were performed for each sample.

## Supplementary Information

**Additional file 1: Figure S1.** Diagram of the crossing strategy to obtain homozygous double knockout strains in *P. xylostella*. **Figure S2.** Diagram of the crossing strategy used for generation of the genetic hybrid strain. **Table S1.** Primers used in this study. **Table S2.** Toxicity to Cry1Ac toxin in larvae from the polygenic knockout strains and their F1 progeny.

**Additional file 2.** The raw data of all the figures and statistical analyses in this study.

## Data Availability

All relevant data are within the manuscript and its Supporting Information files. The raw data of all the figures and statistical analyses in this study are provided in Additional files 2.

## References

[1] Sanahuja G, Banakar R, Twyman RM, Capell T, Christou P (2011). *Bacillus thuringiensis*: a century of research, development and commercial applications. Plant Biotechnol J.

[2] Bravo A, Likitvivatanavong S, Gill SS, Soberón M (2011). *Bacillus thuringiensis*: A story of a successful bioinsecticide. Insect Biochem Mol Biol.

[3] ISAAA (2019). Global status of commercialized biotech/GM crops in 2019: biotech crops drive socio-economic development and sustainable environment in the new frontier. *ISAAA Brief* No. 55.

[4] Tabashnik BE, Brévault T, Carrière Y (2013). Insect resistance to Bt crops: lessons from the first billion acres. Nat Biotechnol.

[5] Tabashnik BE, Carrière Y (2017). Surge in insect resistance to transgenic crops and prospects for sustainability. Nat Biotechnol.

[6] Jurat-Fuentes JL, Heckel DG, Ferré J (2021). Mechanisms of resistance to insecticidal proteins from *Bacillus thuringiensis*. Annu Rev Entomol.

[7] Soberón M, Pardo-López L, López I, Gómez I, Tabashnik BE, Bravo A (2007). Engineering modified Bt toxins to counter insect resistance. Science.

[8] Tabashnik BE, Huang F, Ghimire MN, Leonard BR, Siegfried BD, Rangasamy M, Yang Y, Wu Y, Gahan LJ, Heckel DG, Bravo A, Soberón M (2011). Efficacy of genetically modified Bt toxins against insects with different genetic mechanisms of resistance. Nat Biotechnol.

[9] Carrière Y, Crickmore N, Tabashnik BE (2015). Optimizing pyramided transgenic Bt crops for sustainable pest management. Nat Biotechnol.

[10] Badran AH, Guzov VM, Huai Q, Kemp MM, Vishwanath P, Kain W, Nance AM, Evdokimov A, Moshiri F, Turner KH, Wang P, Malvar T, Liu DR (2016). Continuous evolution of *Bacillus thuringiensis* toxins overcomes insect resistance. Nature.

[11] Tabashnik BE, Liesner LR, Ellsworth PC, Unnithan GC, Fabrick JA, Naranjo SE, Li XC, Dennehy TJ, Antilla L, Staten RT, Carrière Y (2021). Transgenic cotton and sterile insect releases synergize eradication of pink bollworm a century after it invaded the United States. Proc Natl Acad Sci U S A.

[12] Gould F, Brown ZS, Kuzma J (2018). Wicked evolution: can we address the sociobiological dilemma of pesticide resistance?. Science.

[13] Pardo-López L, Soberón M, Bravo A (2013). *Bacillus thuringiensis* insecticidal three-domain Cry toxins: mode of action, insect resistance and consequences for crop protection. FEMS Microbiol Rev.

[14] Pinos D, Andrés-Garrido A, Ferré J, Hernández-Martínez P (2021). Response mechanisms of invertebrates to *Bacillus thuringiensis* and its pesticidal proteins. Microbiol Mol Biol Rev.

[15] Adang MJ, Crickmore N, Jurat-Fuentes JL (2014). Diversity of *Bacillus thuringiensis* crystal toxins and mechanism of action. Adv Insect Physiol.

[16] Wu Y (2014). Detection and mechanisms of resistance evolved in insects to Cry toxins from *Bacillus thuringiensis*. Adv Insect Physiol.

[17] Gahan LJ, Pauchet Y, Vogel H, Heckel DG (2010). An ABC transporter mutation is correlated with insect resistance to *Bacillus thuringiensis* Cry1Ac toxin. PLoS Genet.

[18] Baxter SW, Badenes-Pérez FR, Morrison A, Vogel H, Crickmore N, Kain W, Wang P, Heckel DG, Jiggins CD (2011). Parallel evolution of *Bacillus thuringiensis* toxin resistance in Lepidoptera. Genetics.

[19] Atsumi S, Miyamoto K, Yamamoto K, Narukawa J, Kawai S, Sezutsu H, Kobayashi I, Uchino K, Tamura T, Mita K, Kadono-Okuda K, Wada S, Kanda K, Goldsmith MR, Noda H (2012). Single amino acid mutation in an ATP-binding cassette transporter gene causes resistance to Bt toxin Cry1Ab in the silkworm, *Bombyx mori*. Proc Natl Acad Sci U S A.

[20] Park Y, Gonzalez-Martinez RM, Navarro-Cerrillo G, Chakroun M, Kim Y, Ziarsolo P, Blanca J, Canizares J, Ferre J, Herrero S (2014). ABCC transporters mediate insect resistance to multiple Bt toxins revealed by bulk segregant analysis. BMC Biol.

[21] Xiao Y, Zhang T, Liu C, Heckel DG, Li X, Tabashnik BE, Wu K (2014). Mis-splicing of the ABCC2 gene linked with Bt toxin resistance in *Helicoverpa armigera*. Sci Rep.

[22] Guo Z, Kang S, Chen D, Wu Q, Wang S, Xie W, Zhu X, Baxter SW, Zhou X, Jurat-Fuentes JL, Zhang Y (2015). MAPK signaling pathway alters expression of midgut ALP and ABCC genes and causes resistance to *Bacillus thuringiensis* Cry1Ac toxin in diamondback moth. PLoS Genet.

[23] Banerjee R, Hasler J, Meagher R, Nagoshi R, Hietala L, Huang F, Narva K, Jurat-Fuentes JL (2017). Mechanism and DNA-based detection of field-evolved resistance to transgenic Bt corn in fall armyworm (*Spodoptera frugiperda*). Sci Rep.

[24] Flagel L, Lee YW, Wanjugi H, Swarup S, Brown A, Wang J, et al. Mutational disruption of the ABCC2 gene in fall armyworm, *Spodoptera frugiperda*, confers resistance to the Cry1Fa and Cry1A.105 insecticidal proteins. Sci Rep. 2018;8(1):7255. 10.1038/s41598-018-25491-9 10.1038/s41598-018-25491-9 PMC5940765 29740041

[25] Guo Z, Sun D, Kang S, Zhou J, Gong L, Qin J, Guo L, Zhu L, Bai Y, Luo L, Zhang Y (2019). CRISPR/Cas9-mediated knockout of both the *PxABCC2* and *PxABCC3* genes confers high-level resistance to *Bacillus thuringiensis* Cry1Ac toxin in the diamondback moth, *Plutella xylostella* (L.). Insect Biochem Mol Biol.

[26] Boaventura D, Ulrich J, Lueke B, Bolzan A, Okuma D, Gutbrod O, Geibel S, Zeng Q, Dourado PM, Martinelli S, Flagel L, Head G, Nauen R (2020). Molecular characterization of Cry1F resistance in fall armyworm, *Spodoptera frugiperda* from Brazil. Insect Biochem Mol Biol.

[27] Guo Z, Kang S, Sun D, Gong L, Zhou J, Qin J, Guo L, Zhu L, Bai Y, Ye F, Wu Q, Wang S, Crickmore N, Zhou X, Zhang Y (2020). MAPK-dependent hormonal signaling plasticity contributes to overcoming *Bacillus thuringiensis* toxin action in an insect host. Nat Commun.

[28] Jin M, Yang Y, Shan Y, Chakrabarty S, Cheng Y, Soberon M, Bravo A, Liu K, Wu K, Xiao Y (2021). Two ABC transporters are differentially involved in the toxicity of two *Bacillus thuringiensis* Cry1 toxins to the invasive crop-pest *Spodoptera frugiperda* (J. E. Smith). Pest Manag Sci.

[29] Zhang S, Cheng H, Gao Y, Wang G, Liang G, Wu K (2009). Mutation of an aminopeptidase N gene is associated with *Helicoverpa armigera* resistance to *Bacillus thuringiensis* Cry1Ac toxin. Insect Biochem Mol Biol.

[30] Tiewsiri K, Wang P (2011). Differential alteration of two aminopeptidases N associated with resistance to *Bacillus thuringiensis* toxin Cry1Ac in cabbage looper. Proc Natl Acad Sci U S A.

[31] Coates BS, Sumerford DV, Siegfried BD, Hellmich RL, Abel CA (2013). Unlinked genetic loci control the reduced transcription of aminopeptidase N 1 and 3 in the European corn borer and determine tolerance to *Bacillus thuringiensis* Cry1Ab toxin. Insect Biochem Mol Biol.

[32] Chen Y, Li M, Islam I, You L, Wang Y, Li Z, Ling L, Zeng B, Xu J, Huang Y, Tan A (2014). Allelic-specific expression in relation to *Bombyx mori* resistance to Bt toxin. Insect Biochem Mol Biol.

[33] Wang S, Kain W, Wang P (2018). *Bacillus thuringiensis* Cry1A toxins exert toxicity by multiple pathways in insects. Insect Biochem Mol Biol.

[34] Hardstone MC, Scott JG (2010). A review of the interactions between multiple insecticide resistance loci. Pestic Biochem Physiol.

[35] Kim JH, Moreau JA, Zina JM, Mazgaeen L, Yoon KS, Pittendrigh BR, Clark JM (2018). Identification and interaction of multiple genes resulting in DDT resistance in the 91-R strain of *Drosophila melanogaster* by RNAi approaches. Pestic Biochem Physiol.

[36] Bretschneider A, Heckel DG, Pauchet Y (2016). Three toxins, two receptors, one mechanism: Mode of action of Cry1A toxins from *Bacillus thuringiensis* in *Heliothis virescens*. Insect Biochem Mol Biol.

[37] Tanaka S, Miyamoto K, Noda H, Jurat-Fuentes JL, Yoshizawa Y, Endo H, Sato R (2013). The ATP-binding cassette transporter subfamily C member 2 in *Bombyx mori* larvae is a functional receptor for Cry toxins from *Bacillus thuringiensis*. FEBS J.

[38] Chen Z, He F, Xiao Y, Liu C, Li J, Yang Y, Ai H, Peng J, Hong H, Liu K. Endogenous expression of a Bt toxin receptor in the Cry1Ac-susceptible insect cell line and its synergistic effect with cadherin on cytotoxicity of activated Cry1Ac. Insect Biochem Mol Biol 2015;59(0):1–17, 10.1016/j.ibmb.2015.01.014. 10.1016/j.ibmb.2015.01.014 25662100

[39] Tanaka S, Endo H, Adegawa S, Kikuta S, Sato R (2016). Functional characterization of *Bacillus thuringiensis* Cry toxin receptors explains resistance in insects. FEBS J.

[40] Gratz SJ, Cummings AM, Nguyen JN, Hamm DC, Donohue LK, Harrison MM, Wildonger J, O'Connor-Giles KM (2013). Genome engineering of *Drosophila* with the CRISPR RNA-guided Cas9 nuclease. Genetics.

[41] Sun D, Guo Z, Liu Y, Zhang Y (2017). Progress and prospects of CRISPR/Cas systems in insects and other arthropods. Front Physiol.

[42] Wang H, Shi Y, Wang L, Liu S, Wu S, Yang Y, Feyereisen R, Wu Y (2018). CYP6AE gene cluster knockout in *Helicoverpa armigera* reveals role in detoxification of phytochemicals and insecticides. Nat Commun.

[43] Wang J, Ma H, Zhao S, Huang J, Yang Y, Tabashnik BE, Wu Y (2020). Functional redundancy of two ABC transporter proteins in mediating toxicity of *Bacillus thuringiensis* to cotton bollworm. PLoS Pathog.

[44] Liu Z, Fu S, Ma X, Baxter SW, Vasseur L, Xiong L, Huang Y, Yang G, You S, You M (2020). Resistance to *Bacillus thuringiensis* Cry1Ac toxin requires mutations in two *Plutella xylostella* ATP-binding cassette transporter paralogs. PLoS Pathog.

[45] Zhao S, Jiang D, Wang F, Yang Y, Tabashnik BE, Wu Y (2020). Independent and synergistic effects of knocking out two ABC transporter genes on resistance to *Bacillus thuringiensis* toxins Cry1Ac and Cry1Fa in diamondback moth. Toxins.

[46] Crickmore N (2016). *Bacillus thuringiensis* resistance in *Plutella*—too many trees?. Curr Opin Insect Sci.

[47] Baxter SW, Zhao JZ, Gahan LJ, Shelton AM, Tabashnik BE, Heckel DG (2005). Novel genetic basis of field-evolved resistance to Bt toxins in *Plutella xylostella*. Insect Mol Biol.

[48] Guo Z, Kang S, Zhu X, Wu Q, Wang S, Xie W, Zhang Y (2015). The midgut cadherin-like gene is not associated with resistance to *Bacillus thuringiensis* toxin Cry1Ac in *Plutella xylostella* (L.). J Invertebr Pathol.

[49] Gassmann AJ, Carrière Y, Tabashnik BE (2009). Fitness costs of insect resistance to *Bacillus thuringiensis*. Annu Rev Entomol.

[50] Guo Z, Kang S, Wu Q, Wang S, Crickmore N, Zhou X, Bravo A, Soberón M, Zhang Y (2021). The regulation landscape of MAPK signaling cascade for thwarting *Bacillus thuringiensis* infection in an insect host. PLoS Pathog.

[51] Sivakumar S, Rajagopal R, Venkatesh GR, Srivastava A, Bhatnagar RK (2007). Knockdown of aminopeptidase-N from *Helicoverpa armigera* larvae and in transfected Sf21 cells by RNA interference reveals its functional interaction with *Bacillus thuringiensis* insecticidal protein Cry1Ac. J Biol Chem.

[52] Aroonkesorn A, Pootanakit K, Katzenmeier G, Angsuthanasombat C (2015). Two specific membrane-bound aminopeptidase N isoforms from *Aedes aegypti* larvae serve as functional receptors for the *Bacillus thuringiensis* Cry4Ba toxin implicating counterpart specificity. Biochem Biophys Res Commun.

[53] Endo H, Tanaka S, Imamura K, Adegawa S, Kikuta S, Sato R (2017). Cry toxin specificities of insect ABCC transporters closely related to lepidopteran ABCC2 transporters. Peptides.

[54] Endo H, Tanaka S, Adegawa S, Ichino F, Tabunoki H, Kikuta S, Sato R (2018). Extracellular loop structures in silkworm ABCC transporters determine their specificities for *Bacillus thuringiensis* Cry toxins. J Biol Chem.

[55] Ma Y, Zhang J, Xiao Y, Yang Y, Liu C, Peng R, Yang Y, Bravo A, Soberón M, Liu K (2019). The cadherin Cry1Ac binding-region is necessary for the cooperative effect with ABCC2 transporter enhancing insecticidal activity of *Bacillus thuringiensis* Cry1Ac toxin. Toxins.

[56] Ren XL, Jiang WL, Ma YJ, Hu HY, Ma XY, Ma Y, Li GQ (2016). The *Spodoptera exigua* (Lepidoptera: Noctuidae) ABCC2 mediates Cry1Ac cytotoxicity and, in conjunction with cadherin, contributes to enhance Cry1Ca toxicity in Sf9 cells. J Econ Entomol.

[57] Stevens T, Song S, Bruning JB, Choo A, Baxter SW (2017). Expressing a moth abcc2 gene in transgenic *Drosophila* causes susceptibility to Bt Cry1Ac without requiring a cadherin-like protein receptor. Insect Biochem Mol Biol.

[58] Zhang D, Jin M, Yang Y, Zhang J, Yang Y, Liu K, Soberón M, Bravo A, Xiao Y, Wu K (2021). Synergistic resistance of *Helicoverpa armigera* to Bt toxins linked to cadherin and ABC transporters mutations. Insect Biochem Mol Biol.

[59] Heckel DG (2021). The essential and enigmatic role of ABC transporters in Bt resistance of noctuids and other insect pests of agriculture. Insects.

[60] Ocelotl J, Sánchez J, Gómez I, Tabashnik BE, Bravo A, Soberón M (2017). ABCC2 is associated with *Bacillus thuringiensis* Cry1Ac toxin oligomerization and membrane insertion in diamondback moth. Sci Rep.

[61] Wei J, Zhang Y, An S (2019). The progress in insect cross-resistance among *Bacillus thuringiensis* toxins. Arch Insect Biochem Physiol.

[62] Ferré J, Van Rie J (2002). Biochemistry and genetics of insect resistance to *Bacillus thuringiensis*. Annu Rev Entomol.

[63] Liu YB, Tabashnik BE, Meyer SK, Crickmore N (2001). Cross-resistance and stability of resistance to *Bacillus thuringiensis* toxin Cry1C in diamondback moth. Appl Environ Microbiol.

[64] Gong Y, Wang C, Yang Y, Wu S, Wu Y (2010). Characterization of resistance to *Bacillus thuringiensis* toxin Cry1Ac in *Plutella xylostella* from China. J Invertebr Pathol.

[65] Tabashnik BE, Dennehy TJ, Carrière Y (2005). Delayed resistance to transgenic cotton in pink bollworm. Proc Natl Acad Sci U S A.

[66] Homem RA, Buttery B, Richardson EE, Tan Y, Field LM, Williamson MS, Davies TGE (2020). Evolutionary trade-offs of insecticide resistance-the fitness costs associated with target-site mutations in the nAChR of *Drosophila melanogaster*. Mol Ecol.

[67] Tang JD, Gilboa S, Roush RT, Shelton AM (1997). Inheritance, stability, and lack-of-fitness costs of field-selected resistance to *Bacillus thuringiensis* in diamondback moth (Lepidoptera: Plutellidae) from Florida. J Econ Entomol.

[68] Mathers TC, Chen Y, Kaithakottil G, Legeai F, Mugford ST, Baa-Puyoulet P, Bretaudeau A, Clavijo B, Colella S, Collin O, Dalmay T, Derrien T, Feng H, Gabaldón T, Jordan A, Julca I, Kettles GJ, Kowitwanich K, Lavenier D, Lenzi P, Lopez-Gomollon S, Loska D, Mapleson D, Maumus F, Moxon S, Price DRG, Sugio A, van Munster M, Uzest M, Waite D, Jander G, Tagu D, Wilson ACC, van Oosterhout C, Swarbreck D, Hogenhout SA (2017). Rapid transcriptional plasticity of duplicated gene clusters enables a clonally reproducing aphid to colonise diverse plant species. Genome Biol.

[69] Flatt T, Tu MP, Tatar M (2005). Hormonal pleiotropy and the juvenile hormone regulation of *Drosophila* development and life history. BioEssays.

[70] Schwenke RA, Lazzaro BP, Wolfner MF (2016). Reproduction-immunity trade-offs in insects. Annu Rev Entomol.

[71] Guo Z, Kang S, Zhu X, Xia J, Wu Q, Wang S, Xie W, Zhang Y (2015). Down-regulation of a novel ABC transporter gene (*Pxwhite*) is associated with Cry1Ac resistance in the diamondback moth, *Plutella xylostella* (L.). Insect Biochem Mol Biol.

[72] Guo Z, Gong L, Kang S, Zhou J, Sun D, Qin J, Guo L, Zhu L, Bai Y, Bravo A, Soberón M, Zhang Y (2020). Comprehensive analysis of Cry1Ac protoxin activation mediated by midgut proteases in susceptible and resistant *Plutella xylostella* (L.). Pestic Biochem Physiol.

[73] Liu YB, Tabashnik BE (1997). Inheritance of resistance to the *Bacillus thuringiensis* toxin Cry1C in the diamondback moth. Appl Environ Microbiol.

