# Supplementary material for: A versatile contribution of both aminopeptidases N and ABC transporters to Bt Cry1Ac toxicity in the diamondback moth
Source: BMC Biol. 2022 Feb 4;20:33. doi: 10.1186/s12915-022-01226-1 (PMC8817492; doi:10.1186/s12915-022-01226-1)
Supplement: Supplementary file 1 — Additional file 1: Figure S1. Diagram of the crossing strategy to obtain homozygous double knockout strains in P. xylostella. Figure S2. Diagram of the crossing strategy used for generation of the genetic hybrid strain. Table S1. Primers used in this study. Table S2. Toxicity to Cry1Ac toxin in larvae from the polygenic knockout strains and their F1 progeny. [file 12915_2022_1226_MOESM1_ESM.docx]

**Additional file 1**

**A versatile contribution of both aminopeptidases N and ABC transporters to Bt Cry1Ac toxicity in the diamondback moth**

Dan Sun^1,2†^, Liuhong Zhu^1†^, Le Guo^1†^, Shaoli Wang^1^, Qingjun Wu^1^, Neil Crickmore^3^, Xuguo Zhou^4^, Alejandra Bravo^5^, Mario Soberón^5^, Zhaojiang Guo^1*^ and Youjun Zhang^1*^

^1^Department of Plant Protection, Institute of Vegetables and Flowers, Chinese Academy of Agricultural Sciences, Beijing 100081, China

^2^Guangdong Laboratory for Lingnan Modern Agriculture, Guangdong 510642, China

^3^School of Life Sciences, University of Sussex, Brighton BN1 9QE, UK

^4^Department of Entomology, University of Kentucky, Lexington, Kentucky 40546-0091, USA

^5^Departamento de Microbiología Molecular, Instituto de Biotecnología, Universidad Nacional Autónoma de México, Apdo. Postal 510-3, Cuernavaca, 62250, Morelos, México

^†^These authors contributed equally to this work.

*Corresponding authors:

Zhaojiang Guo and Youjun Zhang, Ph.D. and Professor

Institute of Vegetables and Flowers, Chinese Academy of Agricultural Sciences

Beijing 100081, China

Tel: 86-10-82109518

Fax: 86-10-82109518

E-mail: guozhaojiang@caas.cn (ZG); zhangyoujun@caas.cn (YZ).

**Fig. S1.** Diagram of the crossing strategy to obtain homozygous double knockout strains in *P. xylostella*. CRISPR/Cas9-mediated dual-sgRNA system was utilized to simultaneously introduce mutations in the *PxABCC2*/*PxABCC3* or *PxAPN1*/*PxAPN3a* loci. After microinjection, single-pair crossing was performed to produce G1 progeny. Heterozygous individuals with double gene mutations were sib-mated to generate G2 progeny. Finally, the stable G3 homozygous knockout strains C2-3KO and N1-3aKO were established by crossing the homozygotes with the double gene mutations. For these genotypes, gray columns represent autosomes, the boxes with two different colors above the autosomes represent the simultaneous introduction of double gene mutations.

**Fig. S2.** Diagram of the crossing strategy used for generation of the genetic hybrid strain. Reciprocal crosses between a genome edited adults from C2-3KO and N1-3aKO were performed to generate G1 progeny (aa represents the homozygous alleles for *PxABCC2* and *PxABCC3* deletions, and bb shows the homozygous alleles for *PxAPN1* and *PxAPN3a* mutations). G1 heterozygous adults were sib-crossed to produce G2 progeny and screened subsequently with a diagnostic dose of Cry1Ac protoxin (10 mg/L), which could kill all the susceptible larvae. The genotypes of the remaining surviving larvae were detected by the banding pattern of PCR products with the four primer pairs in *PxABCC2/PxABCC3*, or *PxAPN1*/*PxAPN3a* loci (shown in Fig. 1c and Fig. 2b). Finally, moths with all of the *PxABCC2*, *PxABCC3*, *PxAPN1* and *PxAPN3a* mutations were mated to establish a stable homozygous C-NKO strain in G3. Numbers in brackets represent the number of individuals with the corresponding genotype.

**Table S1.** Primers used in this study

| Purpose | Gene name | Primer name | Primer sequence (5´-3´) | PCR product (bp) | Tm (°C) |
| --- | --- | --- | --- | --- | --- |
| CRISPR sgRNA* | PxABCC2 | CRISPR-ABCC2-F | *GAAATTAATACGACTCACTATAGG***GCTGTGCAACTTCCTGGCCA**GTTTTAGAGCTAGAAATAGC | 124 | 70 |
|  |  | CRISPR-R | AAAAGCACCGACTCGGTGCCACTTTTTCAAGTTGATAACGGACTAGCCTTATTTTAACTTGCTATTTCTAGCTCTAAAAC |  |  |
|  | PxABCC3 | CRISPR-ABCC3-F | *GAAATTAATACGACTCACTATAGG***GTACTACACGGTGGGCATGG**GTTTTAGAGCTAGAAATAGC | 124 | 70 |
|  |  | CRISPR-R | AAAAGCACCGACTCGGTGCCACTTTTTCAAGTTGATAACGGACTAGCCTTATTTTAACTTGCTATTTCTAGCTCTAAAAC |  |  |
|  | PxAPN1 | CRISPR-APN1-F | *GAAATTAATACGACTCACTATAGGG***AGGCCGTGGGTGTACTGCGC**GTTTTAGAGCTAGAAATAGC | 125 | 70 |
|  |  | CRISPR-R | AAAAGCACCGACTCGGTGCCACTTTTTCAAGTTGATAACGGACTAGCCTTATTTTAACTTGCTATTTCTAGCTCTAAAAC |  |  |
|  | PxAPN3a | CRISPR-APN3a-F | *GAAATTAATACGACTCACTATAG***GGACCTCAACATCGCCACTG**GTTTTAGAGCTAGAAATAGC | 123 | 70 |
|  |  | CRISPR-R | AAAAGCACCGACTCGGTGCCACTTTTTCAAGTTGATAACGGACTAGCCTTATTTTAACTTGCTATTTCTAGCTCTAAAAC |  |  |
| Identification of double genes knockout^†^ | C3-C2-1 | 3-2-F | TAGCCGTTAGATATGTAACTCTCAG | 516 | 63 |
|  |  | 2-3-R | CACCATGACCCGACTGGAGG |  |  |
|  | PxABCC3 | 3-18-F | CAAAGAGCCCGGTGTTCG | 441 | 55 |
|  |  | 3-19-R | TGTAGAGAAACACGAAGATGAC |  |  |
|  | PxABCC2 | 2-13-F | CTTACTATCATCACCGGAACCCTCC | 334 | 63 |
|  |  | 2-12-R | CTACATATTCGATGACCCCCTATCG |  |  |
|  | C3-C2-2 | 3-25-F | CATCCAGCAGACGATCCG | 891 | 60 |
|  |  | 2-26-R | CCGGCAGCCACCTCAACTTC |  |  |
|  | N3-N1-1 | 3a-1-F | CAAGGATTCTCATTGCCTTG | 716 | 58 |
|  |  | 1-13-R | GTGCTGACACTGCCTAGC |  |  |
|  | PxAPN3a | 3a-6-F | TGGTTGAGTACTACAAGAGCTGG | 522 | 55 |
|  |  | 3a-7-R | GTGAAGTCGGGCTTGCTC |  |  |
|  | PxAPN1 | 1-5-F | AGAGAGTAGCCAATATAGTGGCC | 332 | 60 |
|  |  | 1-6-R | TCCACGATGAACCGGGTCTC |  |  |
|  | N3-N1-2 | 3a-14-F | GCTGTGATCGCAAATATTCTGG | 1381 | 60 |
|  |  | 1-2-R | GCCTGAATCAGGACCAACACC |  |  |
| qPCR analysis | RPL32 | qL32-F | CCAATTTACCGCCCTACC | 120 | 55 |
|  |  | qL32-R | TACCCTGTTGTCAATACCTCT |  |  |
|  | PxAPN5 | qAPN5-F | GGACGATCAGGCTGTTAA | 160 | 55 |
|  |  | qAPN5-R | TTCCCGAATCTGGTTGTG |  |  |
|  | PxAPN6 | qAPN6-F | CAGCAGGGACGGATAGAA | 146 | 53 |
|  |  | qAPN6-R | ATCGGTAGCAACGAAGTTAA |  |  |
|  | PxABCC1 | qABCC1-F | GGTGGTGCTCATCTGCTACCTCAT | 165 | 55 |
|  |  | qABCC1-R | ATCCTGACACGCTCATCGGTTTT |  |  |

*A specific oligonucleotide encoding a T7 polymerase-binding site (italicized) and the sgRNA target sequence (underlined and bold) of *PxABCC2,* *PxABCC3*, *PxAPN1,* and *PxAPN3a* were designed as the forward primer CRISPR-F, and a common oligonucleotide encoding the remaining sequences of CRISPR-F were designed as the reverse primer CRISPR-R.

^†^To validate the genotypes of the double-mutant individuals in C2-3KO and N1-3aKO strains, four DNA fragments of different sizes, were amplified by the PCR method with four pairs of primers between *PxABCC2* and *PxABCC3* or *PxAPN1* and *PxAPN3a* locus, respectively. For the primer name, the first number or number plus letter represents the specific gene (2 and 3 for *PxABCC2* and *PxABCC3* gene*,* 1 and 3a for *PxAPN1* and *PxAPN3a* gene), and the second number represents for intron or exon. A 516 bp genome DNA fragment was amplified with the primers 3-2-F/2-3-R if *PxABCC2* and *PxABCC3* genes were simultaneously deleted, also, a 716 bp gDNA fragment was amplified with the primers 3a-1-F/1-12-R if *PxAPN1* and *PxAPN3a* genes were simultaneously deleted. The primer pairs of 3-18-F/3-19-R, 2-12-F/2-13, and 3-25-F/2-26-R were used to respectively amplify the gDNA fragments of 441, 334 and 891 bp to detect the double-gene knockout individuals are homozygous or heterozygous in C2-3KO strain. Additionally, the primer pairs of 3a-13-F/3a-14-R, 1-10-F/1-11-R, and 3a-14-F/1-2-R were respectively used to amplify the gDNA fragments of 522, 332 and 1381 bp to detect the double-gene knockout individuals are homozygous or heterozygous in N1-3aKO strain.

**Table S2**. Toxicity to Cry1Ac toxin in larvae from the polygenic knockout strains and their F1 progeny

| Strains* | Survival rate (%)^†^ | Dominance (*h*)^§^ |
| --- | --- | --- |
| DBM1Ac-S | 0 |  |
| C2-3KO | 98 |  |
| N1-3aKO | 96 |  |
| C-NKO | 100 |  |
| DBM1Ac-S × C2-3KO^#^ | 12 | 0.12 |
| DBM1Ac-S × N1-3aKO^#^ | 10 | 0.10 |
| DBM1Ac-S × C-NKO^#^ | 8 | 0.08 |
| C2-3KO × N1-3aKO^‡^ | 4 |  |
| C2-3KO × C-NKO^‡^ | 96 |  |
| N1-3aKO × C-NKO^‡^ | 92 |  |

*Reciprocal crossing (50 males and 50 females) was performed among DBM1Ac-S, C2-3KO, N1-3aKO and C-NKO strains.

^†^The sample size to obtain the survival rate was 50 larvae for each strain and 100 larvae for each F1 progeny.

^§^The dominance parameter *h* was calculated by the survival rate at 10 mg/L of Cry1Ac toxin as (the survival rate of F1 hybrid progeny–the survival rate of DBM1Ac-S) divided by (the survival rate of resistant strain–the survival rate of DBM1Ac-S). As mentioned elsewhere, the *h* value varies from 0 (completely recessive resistance) to 1 (completely dominant resistance).

^#^Toxicity to Cry1Ac toxin in F1 larvae produced by crossing the polygenic knockout strain and the susceptible DBM1Ac-S strain.

^‡^Toxicity to Cry1Ac toxin in F1 larvae produced by crossing the different polygenic knockout strain.
